# Chlorhexidine vaginal preparation versus standard treatment at caesarean section to reduce endometritis and prevent sepsis—a feasibility study protocol (the PREPS trial)

**DOI:** 10.1186/s40814-018-0273-9

**Published:** 2018-06-04

**Authors:** V. Hodgetts Morton, A. Wilson, C. Hewitt, A. Weckesser, N. Farmer, D. Lissauer, P. Hardy, R. K. Morris

**Affiliations:** 1 Institute of Metabolism and Systems Research, College of Medical and Dental Sciences, University of Birmingham, Birmingham, B15 2TT UK; 2 Birmingham Clinical Trials Unit, Institute of Applied Health Sciences, College of Medical and Dental Sciences, University of Birmingham, Birmingham, B15 2TT UK; 3 School of Health and Social Care, Birmingham City University, Birmingham, B15 3TN UK; 4 Birmingham Women’s and Children’s Hospital NHS Foundation Trust, Mindelsohn Way, Birmingham, B15 2TG UK

**Keywords:** Caesarean section, Chlorhexidine, Endometritis, Sepsis, Post-natal infection, Vaginal cleansing, Feasibility

## Abstract

**Background:**

Worldwide caesarean section (CS) delivery is the most common major operation. Approximately 25% of pregnant women undergo a CS in the UK for delivery of their babies. Sepsis and post-natal infection constitute significant maternal mortality and morbidity. Infection following a CS has a number of primary sources including endometritis occurring in 7–17% of women. Sepsis reduction and reduction in antibiotic use have been identified as a national and international priority. The overarching aim of this research is to reduce infectious morbidity from caesarean sections.

**Methods:**

This is a parallel group feasibility randomised controlled trial comparing vaginal cleansing using chlorhexidine gluconate versus no cleansing (standard practice) at CS to reduce infection. Women will be recruited from four National Health Service maternity units. Two hundred fifty women (125 in each arm) undergoing elective or emergency CS, who are aged 16 years and above, and at least 34 weeks pregnant will be randomised. Allocation to treatment will be on a 1:1 ratio. The study includes a qualitative aspect to develop women centred outcomes of wellbeing after delivery.

**Discussion:**

The success of the feasibility study will be assessed by criteria related to the feasibility measurements to ascertain if a larger study is feasible in its current format, needs modification or is unfeasible, and includes recruitment, adherence, follow-up and withdrawal measures.

**Trial registration:**

The PREPS trial has been registered with ISRCTN (ISRCTN 33435996).

## Background

### Justification for participant population

Worldwide caesarean section (CS) delivery is the most common major operation. Approximately 25% of pregnant women undergo a CS in the UK for delivery of their babies. This equates to approximately 171,000 caesarean sections per year in England alone.

Sepsis and post-natal infection constitute significant maternal mortality and morbidity, as well as having significant impact on post-natal recovery and maternal wellbeing. Infection following a CS has numerous primary sources including endometritis, occurring in 7–17% of women; this equates to approximately 27,000 cases of infection per year in England only. Risk factors for endometritis following CS include in labour caesarean section and ruptured membranes with or without vaginal colonisation with group B streptococcus. Sepsis reduction and reduction in antibiotic use have been identified as national and international priorities, improving maternal health and neonatal wellbeing through the facilitation of ongoing breast feeding.

### Justification for intervention

Prophylactic antibiotics at the time of surgery have been demonstrated to be beneficial in a number of large randomised controlled trials (RCT) and continue to reduce infection rates [1]. Current practice of skin preparation [2] is guided by a recent RCT demonstrating the superiority of a chlorhexidine over an iodine-based solution [3]. In addition to skin preparation with an antiseptic solution, cleansing inside the vagina with povidone iodine has been evaluated [4]. A Cochrane review of seven trials randomising 2816 women (2635 analysed) estimated the effects of vaginal cleansing (all with povidone-iodine) on post-caesarean infectious morbidity [4]. Vaginal preparation immediately before caesarean delivery significantly reduced the incidence of post-caesarean endometritis from 8.3% in control groups to 4.3% in vaginal cleansing groups (average risk ratio (RR) 0.45, 95% confidence interval (CI) 0.25 to 0.81, seven trials, 2635 women). The risk reduction was particularly strong for women who were already in labour at the time of the caesarean delivery (7.4% in the vaginal cleansing group versus 13.0% in the control group; RR 0.56, 95% CI 0.34 to 0.95, three trials, 523 women) and for women with ruptured membranes (4.3% in the vaginal cleansing group versus 17.9% in the control group; RR 0.24, 95% CI 0.10 to 0.55, three trials, 272 women [4]). The above would appear an effective and important strategy to reduce morbidity at CS, yet this has not been adopted within obstetric practice internationally and does not feature within the NICE Intrapartum guideline [5]. This is due to concerns with exposure of the fetus to iodine-based substances, concerns with vaginal staining and allergy to iodine. Iodine is a recognised antibacterial agent, but inactivity by the presence of blood [6] limits its use. Thus, there are a number of reasons to believe that vaginal cleansing with chlorhexidine would be an appropriate alternative to povidone iodine. Some studies show greater reduction in skin flora after application of chlorhexidine (0.5 and 4%) [6] compared with povidone-iodine agents. Also, chlorhexidine may have a greater residual activity after application than other preparations and, unlike povidone iodine, it is not inactivated by the presence of blood. Solutions that contain lower concentrations, such as the commonly used chlorhexidine gluconate and acetate (0.05%), are usually well tolerated and may be used for vaginal preparation [6]. With this preparation, there are no reported cases of allergy. There is one small randomised controlled trial comparing povidone iodine with chlorohexidine gluconate for vaginal cleansing at CS. This suggested that chlorohexidine may be superior, and further research was needed [7].

A Cochrane review of cleansing the vagina in normal vaginal delivery with chlorhexidine showed no evidence of an effect on maternal or neonatal infections with low to moderate confidence, although further large scale trials to detect small but clinically important differences were needed. Importantly, no safety concerns for the mother or baby have been identified within these studies [8].

## Methods

### Aims

The overarching aim of this research is to reduce infectious morbidity from caesarean sections. Specific objectives for this feasibility study include:

- To determine appropriate recruitment and randomisation processes
- To assess if women can remain blinded to the trial intervention
- To determine the sample size required for a definitive trial
- To inform if the intervention can be conducted in a multi-centre RCT
- To develop women focused outcome measures and method of data collection
- To assess data collection of clinical outcomes up to 6 weeks
- To assess withdrawals

### Qualitative study design

A qualitative study will inform the outcomes that will be collected on women in the feasibility RCT. Two focus groups will be performed at the lead site (Birmingham Women’s and Children’s Hospital BWCH) of approximately 7 to 10 women in each group who have undergone a CS and would like to contribute to the development of women-centred outcomes.

Women will be recruited via adverts placed on BWCH notice boards and on the post-natal wards, through social media platforms; community midwives will be asked to identify women, as well as hospital midwives if women re-attend and through patient engagement services at BWCH. We will ask women who have had a CS within the previous 6 months to contribute to focus groups on important women-centred outcomes for research into infection and complications following a CS.

The focus groups will be performed prior to commencement of recruitment to the feasibility RCT and thus used to decide on outcomes for inclusion in the feasibility trial that the women feel are important for post-natal quality of life and recovery. All women will have demographic data collected. The focus groups will be recorded and transcribed anonymously. The data will be analysed thematically and managed using the Framework Method [9]. An experienced qualitative researcher will run the focus groups, with the support of a research associate and the clinical research fellow.

From informal discussions with women who have undergone CS, the outcomes are likely to be composed of the following themes:

- Length of midwife follow-up
- Prolonged use of analgesics
- Ability to care for self
- Time to leaving house with family/independently
- Ability to care for a baby

### Feasibility study design

Blinded to women and personnel performing outcome collection, parallel group, feasibility RCT compared vaginal cleansing using chlorhexidine gluconate or acetate versus no cleansing (standard practice) at CS to reduce infection. Allocation to treatment will be on a 1:1 ratio. A feasibility randomised controlled trial design is being undertaken to test the consent and randomisation processes for women requiring a CS, and the follow-up processes up until the immediate post-natal period, to ensure we can overcome the challenges this poses.

### Trial setting

The trial will be undertaken in maternity hospitals: Birmingham Women’s and Children’s Hospital, Birmingham Heartlands Hospital, Shrewsbury and Telford Hospital (West Midlands) and Sunderland Royal Hospital, to ensure the trial can be extrapolated to a larger multi-centre trial.

### Identification of participants

Given recruitment, consent and randomisation processes will differ for emergency and elective CS due to differences in planning for surgery; the processes for each type of surgery will be detailed separately. All women booking at any of the hospitals over the study period and who are greater than 34 weeks pregnant while recruitment is running will be posted a patient information leaflet. This is to ensure that all women are aware of the study before delivery of their baby/babies. This will be accompanied with patient information leaflets and study information being available in antenatal clinics and triage waiting rooms. Study posters will also be displayed in prominent positions through the hospitals.

### Eligibility criteria and co-enrolment

Women will be included in the trial if they are as follows:

- Greater than or equal to 34 weeks pregnant
- Having a CS
- Able to give informed written consent
- Able to receive a telephone interview at 14 and 30 days post-natal
- Aged 16 years or over

Women will be excluded from the trial if they:

- Have a known allergy to chlorhexidine gluconate or any of its ingredients
- Are receiving prophylactic intravenous antibiotics for group B streptococcus (GBS) colonisation
- Are receiving intravenous antibiotics for suspected infection (standard CS intravenous prophylaxis is not an exclusion criteria)
- Are currently enrolled in an RCT for an intervention intended to reduce post-operative surgical site infection

All women already enrolled in an interventional study are permitted to be co-enrolled into PREPS, as long as the intervention is not intended to reduce infection. After consent and randomisation to PREPS, women will not be permitted to enter a further interventional study if the study is evaluating the prevention of infection. Co-enrolment in all observational studies is permitted.

### Randomisation

**Fig. 1:**
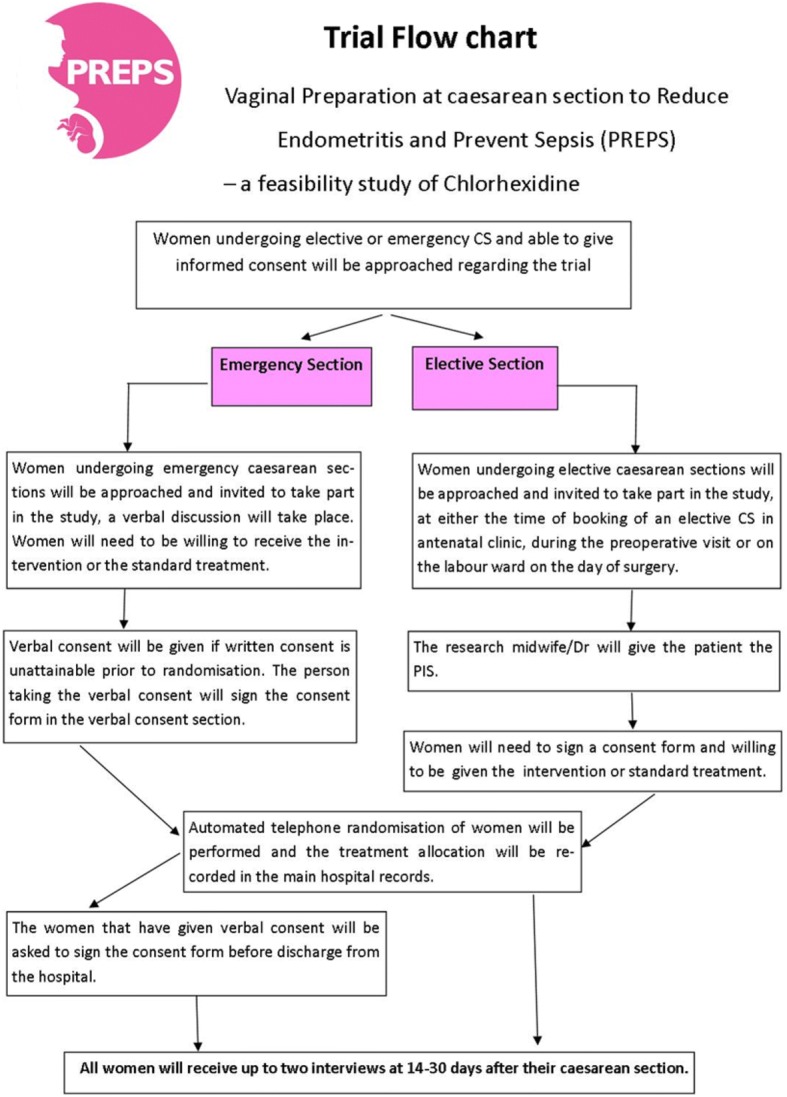
Trial participant flow

After the woman’s eligibility has been confirmed and informed consent has been received, the woman can be randomised into the trial (Fig. 1). Randomisation can be performed by all members of the research team and clinical team and is most likely to be performed by dedicated research midwives.

Randomisation will be provided by a secure automated telephone randomisation system available 24 h a day/7 days a week provided by the University of Aberdeen. The telephone randomisation service will comply with research and governance standards.

The randomised allocation will be documented in the main hospital records on the anaesthetic chart, on a dedicated sticker within the patient notes and on the relevant case report forms. The allocation will not be disclosed to the woman or recorded in the post-natal hand-held notes.

Women will be randomised at the level of the individual in a 1:1 ratio to either chlorohexidine gluconate or acetate 0.05% vaginal cleansing or standard treatment of no vaginal cleansing. A minimisation algorithm will be used to ensure balance in the treatment allocation over the following variables:

- Randomising centre
- In labour and not in labour CS

A ‘random element’ will be included in the minimisation algorithm so that each patient has a probability (unspecified here) of being randomised to the opposite treatment that they would have otherwise received.

### Blinding

The trial cannot be blinded to the operator or the clinical care team in theatre providing care to the women due to the nature of the intervention. Randomisation will be performed by a wide range of staff from theatre runners to consultants. Randomisation may be performed by dedicated research midwives who will also collect outcome data from the notes, but there will be no recording of the allocation in the post-natal notes.

Attempts will be made to blind the women as the intervention will be applied at the time of the catheter insertion, and the woman should not be aware of the application due to anaesthesia.

All data collection from the maternal notes will be blinded since there will only be recording of whether the intervention was applied on the theatre operation note and anaesthetic/operation chart, which are not held in the maternal post-natal notes. As a part of the monitoring process, 10% of medical records will be independently monitored and data collection will be verified by the trial sponsor on-site.

The research midwife conducting the telephone follow-up interviews will be blinded to the treatment group so that there will be no bias in the collection of outcomes. The research midwife will not have an access to the medical records or any data collection forms at the time of telephone interviews.

At the end of the 14-day interview, the midwife will ask the woman whether she feels she received the intervention or not to assess whether blinding of the woman is an achievable aim.

### Intervention

Chlorohexidine gluconate 0.05% or chlorohexidine acetate 0.05% will be used to perform vaginal cleansing. The active ingredient is chlorhexidine 0.05%. This is indicated within the British National Formulary for swabbing in obstetrics.

Fifty millilitres of antiseptic (chlorohexidine gluconate 0.05% or chlorohexidine acetate 0.05%) will be emptied into a sterile pot. A single swab/sponge mounted on a sponge holder soaked in the antiseptic will be used to clean the vagina prior to CS at the time of urinary catheter insertion, for guidance, we suggest the vaginal cleansing should take 30 s. Following the CS procedure, the vagina will be cleaned of excess blood as is standard practice with a dry swab. The application of the intervention is quick and familiar to the doctors performing the surgery due to their experience in gynaecological surgery where it is routine practice. All theatres’ standard operating procedures regarding swabs and instrument counts should be adhered to, to ensure patient safety. At all sites, staff will be asked to record if they have performed vaginal cleansing, and if performed, which antiseptic was used, on the CS operation note within the hospital notes.

## Outcomes

All feasibility outcomes will be assessed overall and by centre. Recruitment will take place for a minimum of 12 weeks and a maximum of 20 weeks, across the four maternity sites.

### Stop-go criteria

The decision to continue to a full trial will be decided by pre-defined stop-go criteria based on the following outcomes:

- The proportion of women randomised into the trial of the 250 recruitment target
- The proportion of women who received their allocated intervention out of all those randomised
- The proportion of women remaining in the trial (i.e. not withdrawn) who successfully complete the planned follow-up process for both the 14- and 30-day telephone interview
- Withdrawal from the study

### Other feasibility outcomes

- The proportion of eligible women approached to take part
- The proportion of women randomised who have an elective/emergency CS
- The proportion of women who are randomised into the PREPS trial with verbal consent from the number of women whom have an emergency CS
- The proportion of women randomised who can successfully identify what treatment they received (i.e. vaginal cleansing or no vaginal cleansing)
- The proportion of complete data for each of the clinical and woman reported outcomes of women randomised
- Time taken to perform the telephone interviews
- Reasons for withdrawal

### Clinical outcomes

The following clinical outcomes will be collected to inform sample size calculations for the main RCT.

#### Development of CDC defined endometritis in the post-natal period (days 0–30)

The development of endometritis meeting the CDC definition, in the post-natal period (day 0 of delivery), is the proposed primary outcome for the full RCT. Endometritis will be defined as per the definitions set out by the US Centre’s for Disease Control and Prevention (Centre’s for Disease Control and Prevention 2017) [10].

#### Clinical diagnosis of endometritis in the post-natal period (days 0–30)

Diagnosis of endometritis by a clinician which does not meet the CDC definition or cannot be verified to meet the definition, e.g. a woman treated in the community for suspected endometritis where it is not feasible to establish that this meets the CDC definition or where the diagnosis does not meet the criteria.

#### Maternal sepsis (days 0–42)

Defined according to the NICE sepsis guideline (released July 2016) [10].

#### Length of hospital stay

The length of hospital stay from randomisation to discharge home or transfer to another hospital following CS or up to 6 weeks after randomisation if not discharged.

#### Readmission to hospital after CS for suspected or confirmed infection (days 0–42)

It is defined as readmission to hospital post-discharge home up until 6 weeks postnatally.

#### Antibiotic prescriptions

These are antibiotics prescribed as an inpatient and hospital prescribed outpatient (days 0–42) and antibiotic prescriptions for suspected/confirmed surgical site infection relating to the woman’s CS (uterine, pelvic, abdominal wound and perineal).

### Requiring level 2 or level 3 critical care

This is defined as requiring level 2 or 3 critical care (or obstetric HDU type care) as a result of an infection up until 6 weeks postnatally (days 0–42).

The endometritis outcomes are collected up until day 30 to be consistent with the CDC definition of endometritis, and the sepsis related outcomes are collected up until 6 weeks to be consistent with the national collection of post-natal sepsis.

### Patient-reported outcomes

These will be determined by the qualitative component of this feasibility study and reported as an outcome of the feasibility study along with summary statistics pre-specified in the statistical analysis plan.

## Participant withdrawal

Informed consent is defined as the process of learning the key facts about a clinical trial before deciding whether or not to participate. It is a continuous and dynamic process, and women should be asked about their ongoing willingness to continue participation with the telephone interviews before the interview questions commence. Women should be aware at the beginning that they can freely withdraw (discontinue participation) from the trial (or part of the trial) at any time.

Types of withdrawal as defined are as follows:

### Before the intervention

The woman would like to withdraw from trial treatment, before the intervention, is applied but is willing to be followed up in accordance with the schedule of assessments and if applicable using any central UK NHS bodies for long-term outcomes (i.e. the woman has agreed that data can be collected and used in the trial analysis).

### After the intervention

The woman would like to withdraw from the trial after the allocation is applied and does not wish to participate in the telephone interview but is willing to be followed up at any visits and if applicable using any central UK NHS bodies for long-term outcomes (i.e. the woman has agreed that data can be collected at standard visits and used in the trial analysis, including data collected as part of long-term outcomes).

### Without follow-up

The woman would like to withdraw from the trial after the allocation is applied and is not willing to be followed up in any way for the purposes of the trial and for no further data to be collected (i.e. only data collected prior to the withdrawal can be used in the trial analysis).

### Complete withdrawal

The woman wishes to withdraw completely (i.e. from trial treatment and all follow-ups) and is not willing to have any of their data, including that already collected, to be used in any future trial analysis.

## Adverse events and serious adverse events

The collection and reporting of adverse events (AEs) will be in accordance with the Research Governance Framework for Health and Social Care and the requirements of the Health Research Authority (HRA). No neonatal AEs or SAEs are required to be reported as the intervention is licenced for use in obstetrics over 34 weeks for swabbing as per the SmPC and has a demonstrated safety record in many trials in neonates above 34 weeks gestation [11].

### Adverse events (AE)

There are certain AEs which are commonly expected in participants as a result of pregnancy and/or CS. As these events are well characterised, it is highly unlikely that this trial will reveal any new safety information relating to this intervention. Thus, there will be no AEs recorded.

### Serious adverse events (SAE)

All events which meet the definition of serious will be collected and recorded in the participant notes and the Case Report Form (CRF). SAEs will, in addition, be reported to the trials office immediately and within 24 h of the principal investigator being made aware of the event. Allergic reaction to chlorohexidine requiring treatment will be considered a serious adverse advent.

Events not considered an SAE in PREPS include:

- Hospitalisation for delivery of the baby
- The development of infection and sepsis in the post-natal period that requires inpatient treatment or prolongation of hospitalisation as this is an outcome of the trial
- Obstetric haemorrhage
- Damage to bowel or bladder during surgery
- Prolonged hospitalisation due to neonatal complications
- Thromboembolic events

All other events not detailed above that meet the definition of a SAE should be reported as detailed below.

SAEs should be reported on an SAE Form. When completing the form, the PI will be asked to define the causality and the severity of the SAE. Causality will be assessed as definitely related, probably related, possibly related, unlikely to be related or unrelated. On becoming aware that a woman has experienced an SAE, the Investigator *or delegate(s)* should report the SAE to their own Trust in accordance with local practice and to the BCTU trials office. On receipt of an SAE form, the Chief Investigator (CI) or delegate(s) will independently determine the seriousness and causality of the SAE. An SAE judged by the PI or CI or delegate(s) to have a reasonable causal relationship with the intervention will be regarded as a related SAE. The causality assessment given by the PI will not be downgraded by the CI or delegate(s). If the CI or delegate(s) disagrees with the PI’s causality assessment, the opinion of both parties will be documented, and where the event requires further reporting, the opinion will be provided with the report. The CI *“or delegate(s)”* will also assess all related SAEs for expectedness. If the event is unexpected (i.e. is not defined in the protocol as an expected event), it will be classified as an unexpected and related SAE. BCTU will report all events categorised as unexpected and related SAEs to the main Research Ethics Committee (REC) within 15 days. The main REC and sponsor will be notified immediately if a significant safety issue is identified during the course of the trial.

## Sample size and data analysis

### Sample size

Since this is a feasibility study, no formal sample size calculations have been undertaken. The feasibility study is not designed or powered to detect a statistically significant difference in efficacy between the two treatment arms. A total sample size of 250 participants would agree with existing literature which suggests that the size of the feasibility study should be at least 10% of the anticipated size of the substantive study [12]. A recruitment target of 250 participants has been chosen for this feasibility study as we expect this number will be sufficient to provide estimates of the feasibility outcomes.

Preliminary sample size calculations were computed for the full RCT. Vaginal cleansing with povidone iodine has been evaluated in a Cochrane review which comprised of seven trials randomising 2816 women (2635 analysed) estimating the effects of vaginal cleansing (povidone-iodine) on post-caesarean infectious morbidity. The risk of bias was generally low, with the quality of most of the studies being high. Vaginal preparation immediately before caesarean delivery significantly reduced the incidence of post-caesarean endometritis from 8.3% in control groups to 4.3% in vaginal cleansing groups. To detect a difference of this size with 90% power and alpha at 5%, we would require 1548 participants. In addition, to account for an anticipated 10% loss to follow-up, we would need a total of 1720 participants.

### Analysis of outcome measures

A separate statistical analysis plan for the PREPS feasibility study will provide a detailed description of the planned statistical analyses. A brief outline of the planned analyses is given below.

All *clinical* outcomes will be analysed according to the treatment arm to which they were randomised (i.e. vaginal cleansing or no cleansing) irrespective of compliance with the randomised treatment allocation, as per the intention to treat principle. Women who did not undergo a CS will be excluded from the analyses.

All *feasibility* outcomes will be analysed pooling the two randomised groups and presenting overall estimates of proportions with 95% confidence intervals, as well as estimates by centre.

All outcomes will primarily take the form of simple descriptive statistics (e.g. proportions and percentages, means and standard deviations) and where appropriate, point estimates of effect sizes (e.g. mean differences and relative risks) and associated 95% confidence intervals.

### Decision to continue to a definitive trial

The decision to continue to a full trial will be decided by pre-defined stop-go criteria. A traffic light system has been designed that will determine progression.

#### Stop-go criteria

- Recruitment rates: the proportion of women randomised into the trial of the 250 recruitment target.
- Adherence to the allocated intervention: the proportion of women who received their allocated intervention out of all those randomised.
- Successful completion of follow-up: the proportion of women remaining in the trial (i.e. not withdrawn as per criteria below) who successfully complete the planned follow-up process for both the 14- and 30-day telephone interview.
- Study drop-out: withdrawal from the study.

#### Traffic light system

Green light: recruitment rate > 90%, adherence rate > 75%, follow-up rate > 90% and dropout rate < 15%. If all four criteria are met, we will proceed to a full trial with the protocol unchanged (unless there is a clear message from the focus groups that would improve the protocol).

Amber light: recruitment rate 80–90%, adherence rate 50–75%, follow-up rate 75–90% or dropout rate 15–30%. If one or more of our amber light criteria are met, we will plan to adapt the protocol in light of the feedback from the focus groups and our experience to improve whichever criteria are not at the ‘green-light’ level before proceeding to full trial. We will assess whether adaption of the protocol will require a further feasibility study or pilot study before progressing.

Red light: recruitment rate < 80%, adherence rate < 50%, follow-up rate < 75% or dropout rate > 30%.

If one or more of these criteria are met, we would consider the current protocol not feasible and not progress to a full RCT.

The Trial Oversight Committee will take into consideration statistical uncertainty around these rates using 95% confidence intervals.

Missing data: Every attempt will be made to collect full follow-up data on all study participants; it is thus anticipated that missing data will be minimal and the strategies needed to achieve this are part of this feasibility RCT. The main analysis will use available data only; however, the amount of missing data will be assessed, and if necessary, sensitivity analyses will be undertaken.

## Trial and data management

### Sponsor

The PREPS trial is sponsored by Birmingham Women’s and Children’s NHS Foundation Trust.

### Coordinating centre

The coordinating centre is the Birmingham Clinical Trials Unit at the University of Birmingham.

### Trial oversight committee

Given this is a feasibility study, the Trial Oversight Committee (TOC) will comprise of a joint trial steering committee and data monitoring committee and will meet three times through the proposed 15-month study. The TOC will provide supervision and advice for the study and ensure the study is conducted as applicable to the MRC Guidelines for Good Clinical Practice in Clinical Trials. Trial data provided to the TOC will be anonymised, but study group allocation may be provided, if it is necessary for their deliberations regarding serious adverse events. There is no planned interim analysis.

### Data management

Processes will be employed to facilitate the accuracy of the data included in the final report. These processes will be detailed in the trial specific data management plan. Coding and validation will be agreed between the trial’s coordinator, statistician and programmer, and the trial database will be signed off once the implementation of these has been assured.

### Data security

The security of the trial database is governed by the policies of the University of Birmingham. The University’s Data Protection Policy and the Conditions of Use of Computing and Network Facilities set out the security arrangements under which sensitive data should be processed and stored. All studies at the University of Birmingham have to be registered with the Data Protection Officer and data held in accordance with the Data Protection Act. The University will designate a Data Protection Officer upon registration of the study. The study centre has arrangements in place for the secure storage and processing of the study data which comply with the University of Birmingham policies.

## Discussion

Post-partum infection/sepsis is a significant global problem. With the increasing evidence regarding antimicrobial resistance and the development of bacterial resistance there, is concern that without action, common procedures such as CS will carry significant risks. We must, therefore, seek strategies that reduce this risk. We aim to perform a feasibility study for a larger multi-centre randomised controlled trial (RCT) comparing vaginal cleansing with chlorhexidine versus standard practice of no vaginal cleansing immediately before CS to reduce post-partum endometritis and sepsis. There are a number of difficulties in performing a RCT in pregnant women undergoing CS, particularly in an emergency procedure where there is a short interval between decision and delivery. Additionally, the follow-up of women post-CS is unlike other surgical procedures: mothers are discharged from obstetric care quickly with no routine post-operative follow-up. They are motivated to recover and care for their baby. It is therefore necessary to perform this feasibility trial to assess both our ability to recruit women and adequately follow them up. The main limitation of this feasibility study is that it is being performed within units motivated to perform the feasibility trial and this may not accurately represent recruitment in all sites.

## Conclusion

This is a feasibility randomised controlled trial, assessing the feasibility of performing a trial of vaginal chlorohexidine cleansing to prevent post-natal infection. Testing newly developed verbal consent, randomisation and follow-up processes in this population.

## Trial status

The PREPS trial opened to recruitment on 11th November 2017. At the time of submitting this protocol for publication, the trial was actively recruiting participants.

## Abbreviations

AEs: Adverse events
BCTU: Birmingham Clinical Trials Unit
BWCH: Birmingham Women’s and Children’s Hospital
CDC: Centre for Disease Control and Prevention
CI: Chief investigator
CRF: Case report form
CS: Caesarean section
HRA: Health Research Authority
NHS: National Health Service
NICE: National Institute for Clinical Excellence
PI: Principal investigator
PIL: Patient information leaflet
RCT: Randomised controlled trial
REC: Research Ethics Committee
RR: Risk ratio
SAE: Serious adverse events
SmPC: Summary of product characteristics
